# Supplementary material for: Eating Perception, Nutrition Knowledge and Body Image among Para-Athletes: Practical Challenges in Nutritional Support
Source: Nutrients. 2021 Sep 6;13(9):3120. doi: 10.3390/nu13093120 (PMC8465964; doi:10.3390/nu13093120)
Supplement: Supplementary file 1 [file nutrients-13-03120-s001.zip › nutrients-1341981-supplementary.pdf]

Appendix:Survey questions

About Yourself

Please fill out the following questions.

Q1. What is your age?

____

Q2. What is your gender?

- o Men
- o Women
- o Do not respond

Q3. Who do you live with?

- o Living alone
- o Living with family or partner
- o Other (please specify) ____________________________________

Q4. Do you have any children?

- o Yes (the number of children) ____________________________________
- o No

Q5. Do you have any caregivers for your support?

- o Yes (relationship with you) ____________________________________
- o No

Q6. What kind of support do you have from your caregiver? Please tick all that apply.

- o Daily shopping
- o Meal preparation
- o Dressing
- o Movement
- o Excretion
- o Other (please specify) ____________________________________
- o Do not apply

Q7. What types of disabilities do you have? Please answer the level of injury if in the case of spinal cord injury.

____

Q8. When did you acquire your disabilities?

- o Congenital
- o Acquired (Age at the onset time, example 2 years and 2 months) __________________________

Q9. Do you have limb defect or amputation?

- o Yes (defect site) ____________________________________
- o No

Q10. Do you use prosthesises (artificial limbs)?

- o Yes (attachment site) ____________________________________
- o No

Q11. Do you use a wheelchair?

- o Yes (electric or manual) ____________________________________
- o No

Q12. Do you use a cane/canes?

- o Yes

- No

Q13. Do you have any bedsores?

- Yes (occurrence site) ______________________________
- No

Q14. What is the main sport you participate in?

(Main sports) ______________________________

(Years of competition, e.q. “2 years and 2 months”) ______________________________

General Nutrition Knowledge

Please fill out the following questions not to be affected by whether you like or dislike the food.
Nutritional knowledge questions with correct responses highlighted

Q15. Are these foods ‘High’ or ‘Low’ in carbohydrate? Please tick one box per food.

	High	Low	Not sure
Beef	○	○	○
Rice	○	○	○
Tofu	○	○	○
Onion	○	○	○

Q16. Do you know what the glycemic index (GI) is?

- Yes
- No

If you answer ‘No’, please go to Q20.

Q17. Which of the following phrases best describes the glycemic index?

- The amount of carbohydrate a food contains
- The extent to which carbohydrate food raises blood sugar levels
- The extent to which protein food raises blood sugar levels
- The extent to which carbohydrate food raises blood pressure
- Not sure

Q18. In general, which carbohydrate is best to eat, with high glycemic index or low glycemic index?

- High
- Low
- Not sure

Q19. Which of these foods are classified as high or low in the glycemic index?

	High	Low	Not sure
Potato	○	○	○
Dark chocolate (>70% cacao)	○	○	○
Plain yogurt	○	○	○
Plain bread	○	○	○

Q20. Are these foods ‘High’ or ‘Low’ in protein? Please tick one box per food.

	High	Low	Not sure
Chicken	○	○	○
Potato	○	○	○
Spinach	○	○	○
Tuna	○	○	○

Q21. For improvements in health, what type of fat do experts recommend shouldbe reduced in the diet?

- o Monosaturated fat
- o Polyunsaturated fat
- o Saturated fat
- o Not sure

Q22. Are these foods 'High' or 'Low' in fat? Please tick one box per food.

	High	Low	Not sure
Orange	o	o	o
Avocado	o	o	o
Peanuts	o	o	o
Butter	o	o	o

Q23. If you want to eat something that is rich in vitamin C, which of the following foods would you eat?

- o Orange
- o Beef
- o Whitebait
- o Milk

Q24. If you want to eat something that is rich in iron, which of the following foods would you eat?

- o Milk
- o Spinach
- o Banana
- o Potato

Q25. If you want to eat something that is rich in calcium, which of the following foods would you eat?

- o Potato
- o Orange
- o Spinach
- o Milk

Q26. During resting conditions, which is the predominant energy source the body uses?

- o Carbohydrate
- o Protein
- o Fat
- o Not sure

Q27. How many calories does 1 gram of each of the following macronutrients and alcohol contain?
Please tick one box per item.

	2 kcal	4 kcal	7 kcal	9 kcal	13 kcal	Not sure
Carbohydrate	o	o	o	o	o	o
Protein	o	o	o	o	o	o
Fat	o	o	o	o	o	o
Alcohol	o	o	o	o	o	o

Q28. In general, how much fluid should you drink per day?

- o 0 – 0.99 L
- o 1 – 1.99 L
- o 2 – 2.99 L
- o 3 – 3.99 L
- o 4 – 4.99 L
- o Not sure

Q29. In general, what percentage of water do you lose from the body to experience symptoms such as dizziness and nauseas?

- 0.5 %
- 2.0 %
- 4.0 %
- 6.0 %
- 8.0 %
- Not sure

Q30. If an amino acid is termed ‘essential’ what does this mean?

- You should consume this in your diet
- It is produced in the body
- Should eat it before you exercise
- Not sure

Q31. Do you think alcohol can make you put on weight?

- Yes
- No
- Not sure

Q32. How much pure alcohol is defined as ‘upper limit of the appropriate alcohol consumption’ by Ministry of Health, Labour,
and Welfare

- 10 g/day
- 20 g/day
- 30 g/day
- Not sure

Sport Nutrition Knowledge

Please fill out the following questions not being affected by whether you like or dislike the food.

Q33. Eating more protein is the most important dietary change if you want to have more muscle.

- Agree
- Disagree
- Not sure

Q34. Eating more energy from protein than you need can make you put on fat.

- Agree
- Disagree
- Not sure

Q35. When we exercise at a low intensity, our body mostly uses fat as a fuel.

- Agree
- Disagree
- Not sure

Q36. The body has a limited ability to use protein for muscle protein synthesis.

- Agree
- Disagree
- Not sure

Q37. Which is closest to the protein needs for a 100 kg well trained resistance athlete?

- 100 g (1 g/kg B.W)
- 150 g (1.5 g/kg B.W)
- 500 g (5 g/kg B.W)
- They should eat as much protein as possible
- Not sure

Q38. A fit person eating a balanced diet can improve their athletic performance by eating more vitamins and minerals from food

- Agree
- Disagree
- Not sure

Q39. What are high in the food that athletes should eat before competition?

- Fluids, fat, and carbohydrate
- Fluids, fiber, and protein
- Fluids and carbohydrate
- Not sure

Q40. Eating carbohydrates when you exercise will help keep blood glucose levels stable.

- Agree
- Disagree
- Not sure

Q41. Vitamin C supplements should always be taken by athletes.

- Agree
- Disagree
- Not sure

Q42. All supplements are tested to make sure they are safe, don't have any contamination.

- Agree
- Disagree
- Not sure

Q43. When glycogen loading (carbohydrate loading), what percentage of your diet should come from carbohydrate?

- 45 %
- 60 %
- 75 %
- 90 %
- Not sure

Q44. Which carbohydrates is best to consume immediately post exercise with high GI or low GI to support muscle glycogen recovery?

- High GI
- Low GI
- Not sure

Body Image

Q45. Please fill out the following questions.

	Item	Never	Seldom	Sometimes	Often	Always
1	I respect my body.	○	○	○	○	○
2	I feel good about my body.	○	○	○	○	○
3	I feel that my body has a least some good qualities.	○	○	○	○	○
4	I take a positive attitude towards my body.	○	○	○	○	○
5	I am attentive to my body's needs.	○	○	○	○	○
6	I feel love for my body.	○	○	○	○	○
7	I appreciate the different and unique	○	○	○	○	○

8	My behavior reveals my positive attitude toward my body; for example, I walk holding my head high and smiling.	○	○	○	○	○
9	I am comfortable in my body.	○	○	○	○	○
10	I feel like I am beautiful even if I am different from media images of attractive people (e.g., models, actresses/ actors).	○	○	○	○	○

Eating behavior

Q46. Please fill out the following questions.

	Item	Never	Seldom	Sometimes	Often	Always
1	I am careful about what to eat for my health.	○	○	○	○	○
2	I measure my body weight.	○	○	○	○	○
3	I eat meals on a regular schedule.	○	○	○	○	○
4	I think about my weight (not to gain weight), when I eat meals.	○	○	○	○	○
5	I serve well-proportioned foods (enough amount) on my plate.	○	○	○	○	○
6	I stop eating, even if I want to eat a little more.	○	○	○	○	○
7	I refrain from eating foods for my body even if I like to eat more.	○	○	○	○	○
8	I include both favorite and non-favorite foods on my diet.	○	○	○	○	○
9	I eat foods on my plate even if there is something I dislike.	○	○	○	○	○

Practice of Sport Nutrition

Please fill out the following questions.

Q47. How do you think about the amount of food and drink you take every day?

	Adequate	Not adequate	Not sure
Food intake	○	○	○
Fluid intake	○	○	○

Q48. Please explain why you answered the previous question in that way.

____

Q49. What is the source of information about nutrition? Please tick all that apply.

- ○ Television
- ○ Internet
- ○ Radio
- ○ Newspaper
- ○ Book
- ○ Coach
- ○ Family
- ○ Friend
- ○ Caregiver
- ○ Doctor
- ○ Dietitian
- ○ Other (please specify) ____________________________

- Do not get information about nutrition

Q50. Do you know what foods you should consume to improve your competitive performance?

- Know
- Roughly know
- Not sure (want to know)
- Not sure (do not want to know)

If you selected 'Not sure (want to know)', please go to Q53.

If you selected 'Not sure (do not want to know)', please go to Q54.

Q51. Are you able to eat the foods you should consume to improve your competitive performance?

- Yes
- No

If you selected 'Yes', please go to Q54.

Q52. What is the reason that you are not able to eat the foods you should consume to improve your competitive performance?

Please tick all that apply.

- Avoid the foods because I don't like them
- Others prepare my meals (I don't cook myself)
- Cannot buy the foods for economic reasons
- Other (please specify) ________________________________

Q53. Please explain why you don't know about the foods you should consume to improve your competitive performance even though you want to know about them. Please also explain how do you think you can get the information?

____

Q54. Are your family members supportive of you practicing healthy lifestyle?

- Supportive
- Rather supportive
- Rather not supportive
- Not Supportive

Q55. Do you have anything to ask dietitians if you have a chance to talk with them?

- Yes (please specify) ________________________________
- No
